# Supplementary material for: LMIC-PRIEST: Derivation and validation of a clinical severity score for acutely ill adults with suspected COVID-19 in a middle-income setting
Source: medRxiv. 2022 Nov 7:2022.11.06.22281986. Preprint. [Version 1] doi: 10.1101/2022.11.06.22281986 (PMC9665341; doi:10.1101/2022.11.06.22281986)

medRxiv preprint doi: https://doi.org/10.1101/2022.11.06.22281986; this version posted November 7, 2022. The copyright holder for this preprint (which was not certified by peer review) is the author/funder, who has granted medRxiv a license to display the preprint in perpetuity.
It is made available under a CC-BY 4.0 International license .

Supplementary Material 1: Population characteristics Omicron period validation cohort

Characteristic	Statistic/level	Adverse outcome	No adverse outcome	Total
	N	2,787 (2%)	137,733 (98%)	140,520
Age (years)	Mean (SD)	48.8 (18.8)	43.2 (17.1)	43.3 (17.2)
	Median (IQR)	46 (33, 64)	40 (29, 56)	41 (29, 56)
	Range	16 to 95	16 to 110	16 to 110
Sex	Male	1,658 (59.5%)	70,056 (50.9%)	71,714 (51%)
	Female	1,129 (40.5%)	67,677 (49.1%)	68,806 (49%)
Comorbidities	Asthma/COPD	474 (17%)	20,755 (15.1%)	21,229 (15.1%)
	Other Chronic respiratory disease	7 (0.3%)	297 (0.2%)	304 (0.2%)
	Diabetes	801 (28.7%)	21,307 (15.5%)	22,108 (15.7%)
	Hypertension	1,008 (36.2%)	36,227 (26.3%)	37,235 (26.5%)
	Immunosuppression (HIV)	488 (17.5%)	24,430 (17.7%)	24,918 (17.7%)
	Heart Disease	912 (32.7%)	24,078 (17.5%)	24,990 (17.8%)
	Pregnant	43 (1.5%)	727 (0.5%)	770 (0.6%)
AVPU	Missing			3,249 (2.3%)
	Alert	1,702 (61.1%)	125,337 (91%)	127,039 (90.4%)
	Voice	96 (3.4%)	1,916 (1.4%)	2,012 (1.4%)
	Confused	180 (6.5%)	5,327 (3.9%)	5,057 (3.9%)
	Pain	201 (7.2%)	975 (0.7%)	1,176 (0.8%)
	Unresponsive	554 (19.9%)	983 (0.7%)	1,537 (2.3%)
Systolic BP (mmHg)	Missing			3,707 (2.6%)
	N	2,688	134,125	136,813
	Mean (SD)	126 (30)	129.7 (25.3)	129.6 (25.4)
	Median (IQR)	122 (107,143)	127 (113,143)	127 (113,143)
	Range	52 to 288	50 to 300	50 to 300
Pulse rate (beats/min)	Missing			3,582 (2.6%)
	N	2,694	134,244	136,938
	Mean (SD)	100 (24.7)	93 (21.1)	93.1 (21.2)
	Median (IQR)	99 (83,115)	92 (78, 106)	92 (78,106)
	Range	12 to 300	10 to 300	10 to 300
Respiratory rate (breaths/min)	Missing			3,571 (2.5%)
	N	2,690	134,259	136,949
	Mean (SD)	19 (5.5)	18.1 (3.6)	18.1 (3.6)
	Median (IQR)	19 (17,22)	18 (16,20)	18 (16,20)
	Range	8 to 60	1 to 60	1 to 60
Oxygen saturation	Missing			8,260 (5.9%)
	N	2,641	129,619	132,260
	Mean (SD)	93.9 (8.1)	96.3 (4.9)	96.2 (5)

medRxiv preprint doi: https://doi.org/10.1101/2022.11.06.22281986; this version posted November 7, 2022. The copyright holder for this preprint (which was not certified by peer review) is the author/funder, who has granted medRxiv a license to display the preprint in perpetuity.
It is made available under a CC-BY 4.0 International license .

	Median (IQR)	96 (93, 99)	97 (96, 99)	97 (95, 99)
	Range	13 to 100	10 to 100	10 to 100
Oxygen administration	Missing			7,910 (5.6%)
	N	2,662	129,948	132,610
	1 (air)	1,516 (57%)	123,044 (94.7%)	124,560 (93.9%)
	2 (40% O2)	58 (2.2%)	2,407 (1.9%)	2,465 (1.9%)
	3 (28% O2)	2 (0.1%)	82 (0.1%)	84 (0.1%)
	4 (Nasal prongs)	1,19 (4.5%)	2,610 (2%)	2,729 (2.1%)
	5 (FM neb)	11 (0.4%)	378 (0.3%)	389 (0.3%)
	6 (rebreather mask)	1,10 (4.1%)	1,315 (1%)	1,425 (1.1%)
	7 (nasal prongs and rebreather mask)	17 (0.6%)	112 (0.1%)	1,29 (0.1%)
	8 intubated	776 (29.2%)	0	776 (0.6%)
	9 NIV	53 (2%)	0	53 (0.04%)
Temperature (°C)	Missing			3,258 (2.3%)
	N	2,733	134,529	137,262
	Mean (SD)	36.3 (1.1)	36.3 (0.7)	36.3 (0.7)
	Median (IQR)	36.3 (35.9, 36.7)	36.3 (36, 36.6)	36.3 (36, 36.6)
	Range	25 to 40	25 to 41.9	25 to 41.9
Cough	Missing			93,962 (30.8%)
	Present	80 (2.9%)	3,500 (2.5%)	3,580 (2.6%)
Fever	Missing			41,524 (29.6%)
	Present	25 (0.9%)	1,169 (0.9%)	1,194 (0.9%)
COVID PCR	Positive	2,119 (76%)	26,485 (19.2%)	28,604 (20.4%)
Hospital admission	ICU	6,77 (24.3%)	0	6,77 (0.5%)
Death	Within 30 days contact	1,431 (51.4%)	0	1,431 (1%)

medRxiv preprint doi: https://doi.org/10.1101/2022.11.06.22281986; this version posted November 7, 2022. The copyright holder for this preprint (which was not certified by peer review) is the author/funder, who has granted medRxiv a license to display the preprint in perpetuity.
It is made available under a CC-BY 4.0 International license .

Supplementary Material 2: Population characteristics UK PRIEST validation cohort

Characteristic	Statistic/level	Adverse outcome	No adverse outcome	Total
	N	4,579 (22.1%)	16,119 (77.9%)	20,698
Age (years)*	16-49	369 (8.1%)	5,256 (32.6%)	5,625 (27.2%)
	50-65	981 (21.4%)	4,186 (26%)	5,167 (25%)
	66-80	1,527 (33.4%)	3,727 (23.1%)	5,254 (25.4%)
	>80	1,702 (37.2%)	2,950 (18.3%)	4,652 (22.5%)
Sex	Male	2,661 (58.1%)	7,540 (46.8%)	10,201 (49.3%)
	Female	1,918 (41.9%)	8,579 (53.2%)	10,497 (50.7%)
Comorbidities	Asthma	556 (12.1%)	2,820 (17.5%)	3,376 (16.3%)
	Other Chronic respiratory disease	1,045 (22.8%)	2,693 (16.7%)	3,738 (18.1%)
	Diabetes	1,274 (27.8%)	2,816 (17.5%)	4,090 (19.8%)
	Hypertension	1,828 (39.9%)	4,538 (28.2%)	6,366 (30.8%)
	Immunosuppression	171 (3.7%)	456 (2.8%)	627 (3%)
	Heart Disease	912 (32.7%)	24,078 (17.5%)	4,661 (22.5%)
	Pregnant	6 (0.1%)	79 (0.5%)	85 (0.4%)
AVPU	Missing			2,063 (10%)
	Alert	3,030 (66.2%)	13,335 (82.9%)	16,385 (79.2%)
	Voice	263 (5.7%)	234 (1.5%)	497 (2.4%)
	Confused	557 (12.2%)	907 (5.6%)	1,464 (7.1)
	Pain	114 (2.5%)	65 (0.4%)	179 (0.9%)
	Unresponsive	77 (1.7%)	33 (0.2%)	1,10 (0.6%)
Systolic BP (mmHg)	Missing			585 (2.8%)
	N	4,453	15,660	20,113
	Mean (SD)	130.1 (26.7)	135.9 (24.2)	134.6 (24.9)
	Median (IQR)	129 (112,147)	134 (120, 150)	133 (118, 149)
	Range	47 to 254	37 to 264	37 to 264
Pulse rate (beats/min)	Missing			426 (2.1%)
	N	4,485	15,787	20,272
	Mean (SD)	98.3 (23.3)	93.9 (20.9)	94.9 (21.5)
	Median (IQR)	97 (83,112)	92 (80, 107)	93 (80, 108)
	Range	8 to 209	11 to 220	11 to 220
Respiratory rate (breaths/min)	Missing			536 (2.6%)
	N	4,468	15,694	20,162
	Mean (SD)	27.1 (8.5)	22.2 (6.1)	23.3 (7)
	Median (IQR)	25 (21,32)	20 (18,24)	22 (18,26)
	Range	6 to 99	5 to 99	5 to 99
Oxygen saturation	Missing			254 (1.2%)
	N	4,520	15,924	20,444

medRxiv preprint doi: https://doi.org/10.1101/2022.11.06.22281986; this version posted November 7, 2022. The copyright holder for this preprint (which was not certified by peer review) is the author/funder, who has granted medRxiv a license to display the preprint in perpetuity.
It is made available under a CC-BY 4.0 International license .

	Mean (SD)	91.5 (8.8)	95.6 (5.8)	94.7 (6.8)
	Median (IQR)	84 (89, 96)	97 (95, 98)	96 (94, 98)
	Range	22 to 100	13 to 100	13 to 100
Oxygen administration	On Oxygen	1,964 (42.9%)	2,249 (14%)	4,213 (20.4%)
Temperature (°C)	Missing			651 (2.3%)
	N	4,409	15,638	20,047
	Mean (SD)	37.3 (1.2)	37.1 (1)	37.1 (1.1)
	Median (IQR)	37.2 (36.5, 38.2)	36.9 (36.4, 37.7)	37 (36.4, 37.8)
	Range	31.3 to 41.3	25.9 to 42.1	25.9 to 42.1
Cough	Present	2,659 (58.1%)	10,211 (63.4%)	12,870 (62.2%)
Fever	Present	2,271 (49.6%)	7,916 (49.1%)	10,187 (49.2%)
Clinical impression	COVID	3,419 (77.9%)	10,518 (68.5%)	13,937 (70.6%)
Organ Support	Any	2,046 (44.7%)	0	2,046 (9.9%)
Death	Within 30 days contact	3,222 (70.4%)	0	3,222 (15.6%)

*Due to the small number of patients with some individual ages, age was categorised before receipt of the data to ensure anonymity

Supplementary Material 3: Categorisation of continuous variables using TEWS

**Score**	**3**	**2**	**1**	**0**	**1**	**2**	**3**
**Respiratory Rate**		<9		9-14	15-20	21-29	>29
**Pulse Rate**		<41	41-50	51-100	101-110	111-129	>129
**Systolic BP**	≤70	71-80	81-100	101-199		>199	
**Temperature**		<35		35-38.4		≥38.5	
**Neuro**		Confused		Alert	Reacts to Voice	Reacts to pain	Unresponsive

medRxiv preprint doi: https://doi.org/10.1101/2022.11.06.22281986; this version posted November 7, 2022. The copyright holder for this preprint (which was not certified by peer review) is the author/funder, who has granted medRxiv a license to display the preprint in perpetuity.
It is made available under a CC-BY 4.0 International license .

Supplementary Material 4: Multivariable Analysis complete case analysis (N=102, 402)

**Lasso variable selection (unrestricted) (Continuous variables modelled using fractional polynomials)**		
C-statistic: 0.868		
CITL:-0.014		
**Parameter**	**Coefficient**	
	**Unstandardised**	**Standardised**
Age	0.036	0.617
No Supplemental Oxygen	-1.433	-0.379
(Saturation/10) ^3 -897.3	-0.003	-0.369
No Diabetes	-0.559	-0.121
(Temperature/10) ^3 -48.11	0.051	0.159
No Heart Disease	-0.318	-0.121
(AVCPU+1) ^3-1.4*	0.011	0.119
Systolic Blood Pressure	-0.098	-0.004
ln(respiratory rate/10) -0.60	0.502	0.095
Heart Rate	0.004	0.081
Male	0.133	0.066
No Hypertension	0.126	0.056
Not Pregnant	-0.546	-0.046
No Immunosuppression (HIV)	-0.103	-0.039
No Cough	0.128	0.026
No Other chronic lung disease	-0.191	-0.008
No Fever	-0.043	-0.005
Constant	-2.993	-4.248
**Lasso variable selection (unrestricted) (Continuous variables modelled using TEWS categories)**		
C-statistic: 0.863		
CITL:-0.045		
**Parameter**	**Coefficient**	
	**Unstandardised**	**Standardised**
Age	0.034	0.591
No Supplemental Oxygen	-1.433	-0.393

medRxiv preprint doi: https://doi.org/10.1101/2022.11.06.22281986; this version posted November 7, 2022. The copyright holder for this preprint (which was not certified by peer review) is the author/funder, who has granted medRxiv a license to display the preprint in perpetuity.
It is made available under a CC-BY 4.0 International license .

Saturation (point increase TEWS)	0.474-	0.424
No Diabetes	-0.638	-0.241
AVCPU (point increase TEWS)	0.294	0.133
Respiratory Rate (point increase TEWS)	0.168	0.097
No Hypertension	0.184	0.082
Heart Rate (point increase TEWS)	0.089	0.081
No Heart Disease	-0.196	-0.074
Not Pregnant	-0.527	-0.045
Male	0.08	0.04
No Immunosuppression (HIV)	-0.075	-0.028
No Cough	0.124	0.025
Temperature (point increase TEWS)	0.043	0.024
No Other chronic lung disease	-0.367	-0.015
Systolic Blood Pressure (point increase TEWS)	-0.029	-0.012
No Fever	0.059	0.007
Constant	-3.518	-4.229

*AVCPU coded alert=0, Voice=1, Confusion=2, Pain= 3, Unresponsive =4

medRxiv preprint doi: https://doi.org/10.1101/2022.11.06.22281986; this version posted November 7, 2022. The copyright holder for this preprint (which was not certified by peer review) is the author/funder, who has granted medRxiv a license to display the preprint in perpetuity.
It is made available under a CC-BY 4.0 International license .

Supplementary Material 5: Calibration plots split internal validation complete case analysis

i) Unrestricted (continuous variables modelled fractional polynomials)

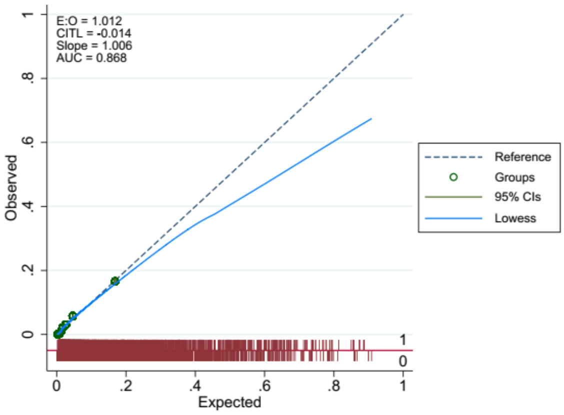

ii) Restricted (continuous variables modelled fractional polynomials)

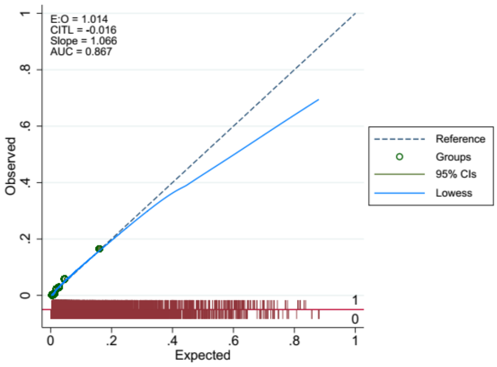

iii) Unrestricted Lasso (continuous variables categorised)

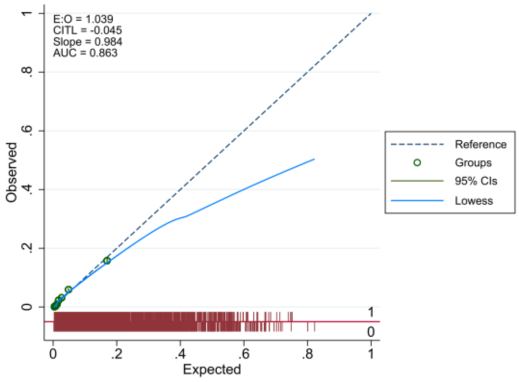

iv) Restricted Lasso (continuous variables categorised)

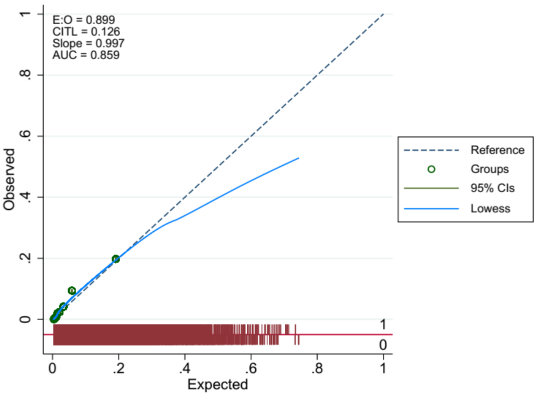

medRxiv preprint doi: https://doi.org/10.1101/2022.11.06.22281986; this version posted November 7, 2022. The copyright holder for this preprint (which was not certified by peer review) is the author/funder, who has granted medRxiv a license to display the preprint in perpetuity.
It is made available under a CC-BY 4.0 International license .

Supplementary Material 6: Unrestricted Deterministic Imputation (N=152,782)

**Lasso variable selection (unrestricted) (Continuous variables modelled using TEWS categories)**		
C-statistic: 0.850		
CITL=-0.018		
**Parameter**	**Coefficient**	
	**Unstandardised**	**Standardised**
Age	0.0027	0.467
No Supplemental Oxygen	-1.696	-0.475
Saturation (point increase TEWS)	0.380	0.357
No Diabetes	-0.638	-0.232
AVCPU (point increase TEWS)	0.294	0.297
Respiratory Rate (point increase TEWS)	0.168	0.058
No Hypertension	0.184	0.066
Heart Rate (point increase TEWS)	0.089	0.062
No Heart Disease	-0.196	-0.041
Not Pregnant	-0.527	-0.037
Male	0.08	0.072
No Immunosuppression (HIV)	-0.075	0.019
No Cough	0.124	0.034
Temperature (point increase TEWS)	0.043	0.044
No Other chronic lung disease	-0.367	-0.019
Systolic Blood Pressure (point increase TEWS)	-0.029	-0.016
No Fever	0.059	0.002
Constant	-2.893	-3.82

medRxiv preprint doi: https://doi.org/10.1101/2022.11.06.22281986; this version posted November 7, 2022. The copyright holder for this preprint (which was not certified by peer review) is the author/funder, who has granted medRxiv a license to display the preprint in perpetuity.
It is made available under a CC-BY 4.0 International license .

Supplementary Material 7: Calibration plots split internal validation deterministic imputation

i) Unrestricted

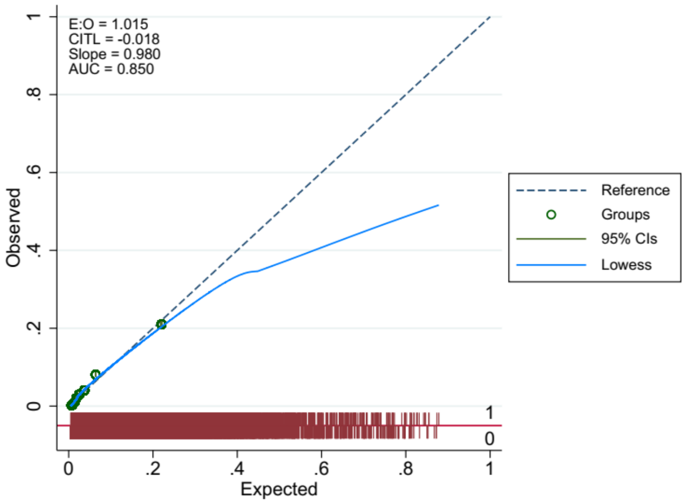

ii) Restricted

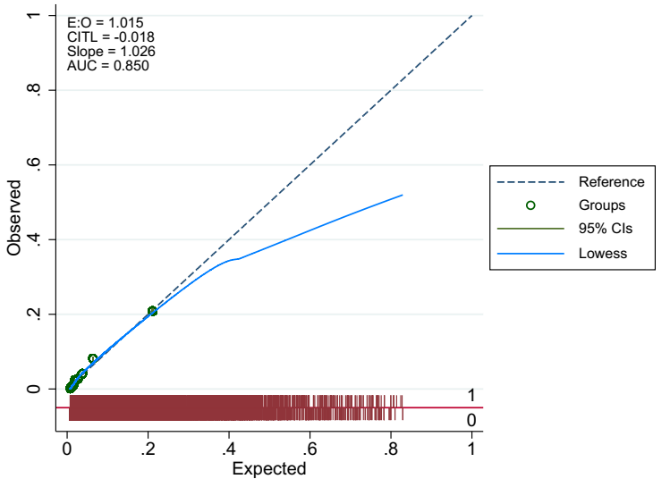

medRxiv preprint doi: https://doi.org/10.1101/2022.11.06.22281986; this version posted November 7, 2022. The copyright holder for this preprint (which was not certified by peer review) is the author/funder, who has granted medRxiv a license to display the preprint in perpetuity.
It is made available under a CC-BY 4.0 International license .

Supplementary Material 8: Multivariable analysis, using multiple imputation (10 imputations; N=152,782)

**Lasso variable selection (unrestricted) (Continuous variables modelled using fractional polynomials)**			
C-statistic: 0.87			
CITL: -0.15			
**Parameter**	**Average coefficient**		**Number times selected**
	**Unstandardised**	**Standardised**	
(Age/10) ^2	0.027	0.463	10
No Supplemental Oxygen	-1.691	-0.486	10
(Saturation/10) ^3	-0.003	-0.348	10
No Diabetes	-0.5778	-0.225	10
(Temperature/10) ^3	0.034	0.114	10
No Heart Disease	-0.123	-0.048	10
(AVCPU+1) ^3	0.019	0.295	10
(Systolic Blood Pressure/100) ^3	-0.032	-0.054	10
ln(respiratory rate/10)	0.034	0.113	10
Heart Rate	0.003	0.073	10
Male	0.165	0.082	10
No Hypertension	0.109	0.049	10
Not Pregnant	-0.435	-0.035	10
No Immunosuppression (HIV)	0.059	0.02	9
No Cough	0.132	0.026	10
No Other chronic lung disease	-0.366	-0.018	10
No Fever	0.055	0.007	8
Constant	-2.117	-3.88	
**Lasso variable selection (unrestricted) (Continuous variables modelled using TEWS categories)**			
C-statistic: 0.864			

medRxiv preprint doi: https://doi.org/10.1101/2022.11.06.22281986; this version posted November 7, 2022. The copyright holder for this preprint (which was not certified by peer review) is the author/funder, who has granted medRxiv a license to display the preprint in perpetuity.
It is made available under a CC-BY 4.0 International license .

CITL: -0.015			
**Parameter**	**Coefficient**		**Number of times**
	**Unstandardised**	**Standardised**	
Age	0.025	0.441	10
No Supplemental Oxygen	-1.71	-0.492	10
Saturation (point increase TEWS)	0.409	0.394	10
No Diabetes	-0.57	-0.222	10
AVCPU (point increase TEWS)	0.41	0.296	10
Respiratory Rate (point increase TEWS)	0.195	0.118	10
No Hypertension	0.134	0.06	10
Heart Rate (point increase TEWS)	0.076	0.070	10
No Heart Disease	-0.102	-0.04	10
Not Pregnant	-0.473	-0.038	10
Male	0.136	0.068	10
No Immunosuppression (HIV)	0.067	0.025	10
No Cough	0.111	0.023	10
Temperature (point increase TEWS)	0.1	0.057	10
No Other chronic lung disease	-0.346	-0.017	10
Systolic Blood Pressure (point increase TEWS)	-0.032	-0.014	10
No Fever	0.003	0.0003	9
Constant	-3.005	-3.888	

medRxiv preprint doi: https://doi.org/10.1101/2022.11.06.22281986; this version posted November 7, 2022. The copyright holder for this preprint (which was not certified by peer review) is the author/funder, who has granted medRxiv a license to display the preprint in perpetuity.
It is made available under a CC-BY 4.0 International license .

Supplementary Material 9: ROC curves for predicting secondary outcome death for LMIC-PRIEST score

i) Development cohort (N=305,564) C stat 0.8346 (95% CI 0.83081 to 0.83835)

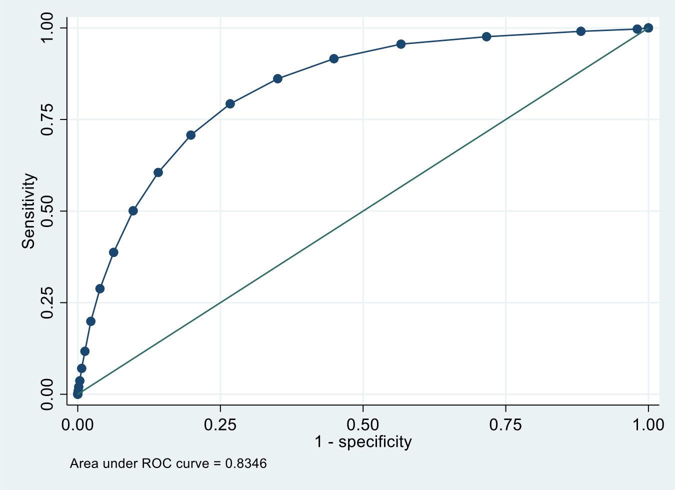

ii) Omicron Validation cohort (N=140,520) C-stat 0. 0.8208 (95% CI: 0.81133 to 0.83025)

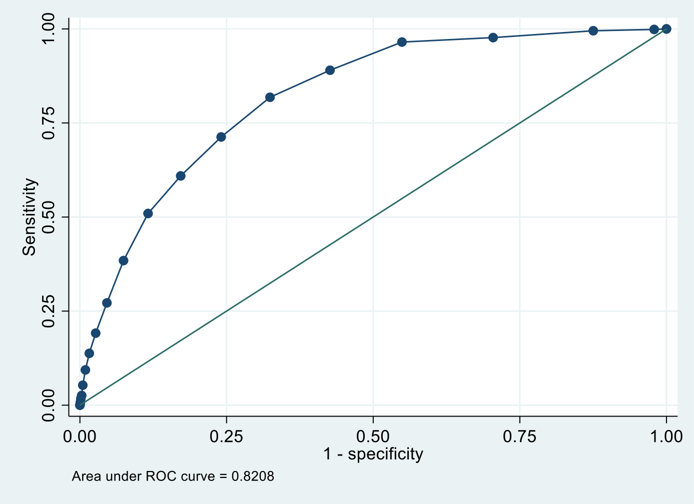

iii) UK PRIEST Validation cohort (N=20,695) C-stat 0.7923 (95%CI: 0.78561 to 0.79903)

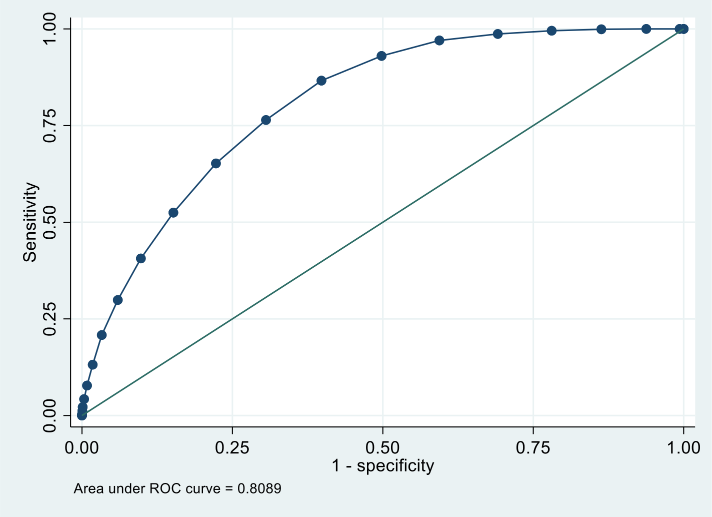

medRxiv preprint doi: https://doi.org/10.1101/2022.11.06.22281986; this version posted November 7, 2022. The copyright holder for this preprint (which was not certified by peer review) is the author/funder, who has granted medRxiv a license to display the preprint in perpetuity.
It is made available under a CC-BY 4.0 International license .

Supplementary Material 10: ROC curves for predicting secondary outcome ICU admission/organ support for LMIC-PRIEST Score

i) Development cohort (N=305,564) C stat 0.7361 (95% CI 0.72403 to 0.74822)

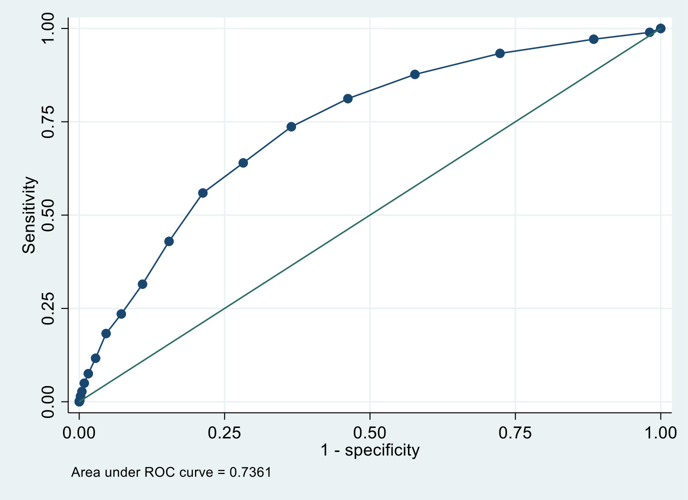

ii) Omicron Validation cohort (N=140,520) C-stat 0.6765 (95% CI: 0.65797 to 0.69506)

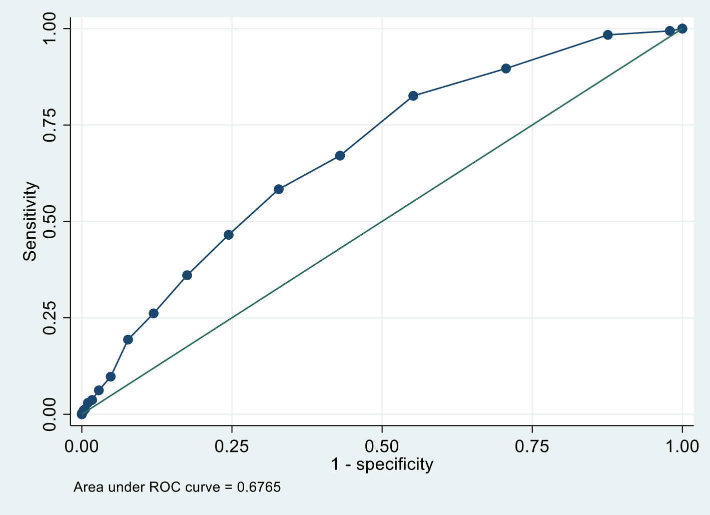

iii) UK PRIEST validation cohort (20,965) C-stat 0.7019 (95% CI: 0.69157 to 0.71216)

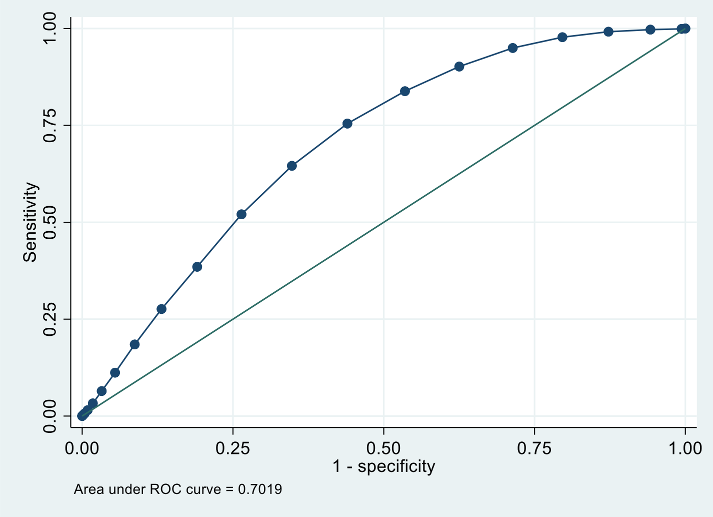

medRxiv preprint doi: https://doi.org/10.1101/2022.11.06.22281986; this version posted November 7, 2022. The copyright holder for this preprint (which was not certified by peer review) is the author/funder, who has granted medRxiv a license to display the preprint in perpetuity.
It is made available under a CC-BY 4.0 International license .

Supplementary Material 11: Sensitivity, specificity, PPV, NPV and proportion with a positive score at each LMIC-PRIEST score threshold for predicting the primary outcome model development cohort (N=305,564)

	Proportion with score	Sensitivity	Specificity	NNP	PPV
>0	98.1%	0.997 (0.996,0.998)	0.02 (0.019,0.02)	0.993 (0.99,0.995)	0.042 (0.041,0.043)
>1	88.5%	0.99 (0.989,0.992)	0.119 (0.118,0.121)	0.997 (0.996,0.997)	0.046 (0.045,0.047)
>2	72.5%	0.974 (0.972,0.977)	0.286 (0.285,0.288)	0.996 (0.996,0.997)	0.056 (0.055,0.057)
>3	57.9%	0.949 (0.945,0.952)	0.437 (0.435,0.439)	0.995 (0.995,0.995)	0.068 (0.067,0.069)
>4	46.4%	0.904 (0.899,0.91	0.555 (0.553,0.557)	0.993 (0.992,0.993)	0.081 (0.079,0.082)
>5	36.7%	0.846 (0.84,0.853)	0.654 (0.652,0.656)	0.99 (0.99,0.99)	0.095 (0.094,0.097)
>6	28.4%	0.773 (0.765,0.780)	0.737 (0.736,0.739)	0.987 (0.986,0.987)	0.112 (0.11,0.114)
>7	21.4%	0.679 (0.671,0.687)	0.806 (0.804,0.807)	0.983 (0.983,0.984)	0.131 (0.128,0.133)
>8	15.6%	0.57 (0.561,0.578)	0.862 (0.861,0.863)	0.979 (0.978,0.98)	0.151 (0.148,0.154)
>9	11%	0.462 (0.453,0.470)	0.905 (0.904,0.906)	0.975 (0.974,0.976)	0.173 (0.169, 0.177)
>10	7.3%	0.353 (0.345,0.362)	0.939 (0.938, 0.940)	0.971 (0.971,0.972)	0.199 (0.194,0.205)
>11	5.7%	0.261 (0.254,0.269)	0.962 (0.962,0.963)	0.968 (0.967,0.969)	0.23 (0.223,0.237)
>12	2.9%	0.181 (0.174,0.187)	0.978 (0.977,0.979)	0.965 (0.965,0.966)	0.261 (0.252,0.27)
>13	1.6%	0.108 (0.103,0.114)	0.988 (0.988, 0.988)	0.963 (0.962, 0.963)	0.281 (0.268, 0.294)
>14	0.9%	0.067 (0.062, 0.071)	0.994 (0.993, 0.994)	0.961 (0.96, 0.962)	0.309 (0.291, 0.327)
>15	0.5%	0.036 (0.033, 0.04)	0.997 (0.996,0.997)	0.96 (0.959, 0.961)	0.317 (0.293, 0.342)
>16	0.2%	0.019 (0.017, 0.021)	0.998 (0.998,0.999)	0.959 (0.959, 0.960)	0.336 (0.301,0.372
>17	0.1%	0.009(0.008, 0.011)	0.999 (0.999, 0.999)	0.959 (0.958, 0.96)	0.326 (0.278, 0.377)

medRxiv preprint doi: https://doi.org/10.1101/2022.11.06.22281986; this version posted November 7, 2022. The copyright holder for this preprint (which was not certified by peer review) is the author/funder, who has granted medRxiv a license to display the preprint in perpetuity.
It is made available under a CC-BY 4.0 International license .

Supplementary Material 12: Sensitivity, specificity, PPV, NPV and proportion with a positive score at each LMIC-PRIEST score threshold for predicting the primary outcome model Omicron validation cohort (N= N=140,520)

	Proportion with score	Sensitivity	Specificity	NNP	PPV
>0	97.9%	0.998 (0.995,0.999)	0.021 (0.02,0.022)	0.998 (0.996,0.999)	0.02 (0.02,0.021)
>1	87.7%	0.995 (0.991,0.997)	0.126 (0.124,0.128)	0.999 (0.999,1)	0.022 (0.022,0.023)
>2	70.7%	0.962 (0.954,0.969)	0.298 (0.295,0.3)	0.997 (0.997,0.998)	0.027 (0.026,0.028)
>3	55.3%	0.929 (0.918,0.938)	0.454 (0.452,0.457)	0.997 (0.996,0.997)	0.033 (0.032,0.035)
>4	43.1%	0.841 (0.827,0.854)	0.577 (0.575,0.58)	0.994 (0.994,0.995)	0.039 (0.037,0.04)
>5	32.9%	0.766 (0.75,0.782)	0.68 (0.677,0.682)	0.993 (0.993,0.994)	0.046 (0.044,0.048)
>6	24.6%	0.663 (0.645,0.68)	0.763 (0.761,0.765)	0.991 (0.991,0.992)	0.054 (0.051,0.056)
>7	17.6%	0.555 (0.537,0.574)	0.831 (0.829,0.833)	0.989 (0.989,0.99)	0.063 (0.06,0.066)
>8	12%	0.443 (0.425,0.462)	0.886 (0.885,0.888)	0.987 (0.987,0.988)	0.073 (0.069,0.077)
>9	11%	0.339 (0.321,0.357)	0.928 (0.926,0.929)	0.986 (0.985,0.986)	0.087 (0.081, 0.092)
>10	4.9%	0.238 (0.222,0.254)	0.955 (0.954, 0.957)	0.984 (0.983,0.985)	0.098 (0.091,0.105)
>11	2.9%	0.159 (0.146,0.173)	0.974 (0.973,0.975)	0.983 (0.982,0.984)	0.111 (0.101,0.121)
>12	1.7%	0.115 (0.103,0.127)	0.985 (0.984,0.985)	0.982 (0.981,0.983)	0.132 (0.119,0.146)
>13	1%	0.076 (0.067, 0.087)	0.991 (0.991, 0.992)	0.981 (0.981, 0.982)	0.148 (0.13, 0.168)
>14	0.5%	0.044 (0.037, 0.052)	0.995 (0.995, 0.996)	0.981 (0.98, 0.982)	0.162 (0.136, 0.19)
>15	0.3%	0.027 (0.022, 0.034)	0.997 (0.997,0.998)	0.981 (0.98, 0.981)	0.166 (0.133, 0.204)
>16	0.2%	0.016 (0.012, 0.022)	0.998 (0.998,0.999)	0.98 (0.98, 0.981)	0.179 (0.133,0.232)
>17	0.1%	0.006 (0.004, 0.01)	0.999 (0.999, 0.999)	0.98 (0.98, 0.98.1)	0.157 (0.095, 0.24)

medRxiv preprint doi: https://doi.org/10.1101/2022.11.06.22281986; this version posted November 7, 2022. The copyright holder for this preprint (which was not certified by peer review) is the author/funder, who has granted medRxiv a license to display the preprint in perpetuity.
It is made available under a CC-BY 4.0 International license .

Supplementary Material 13: Sensitivity, specificity, PPV, NPV and proportion with a positive score at each LMIC-PRIEST score threshold for predicting the primary outcome model UK PRIEST validation cohort (N= N=20,698)

	Proportion with score	Sensitivity	Specificity	NNP	PPV
>0	99.4%	1 (0.998,1)	0.007 (0.006,0.008)	0.983 (0.94,0.998)	0.22 (0.22,0.23)
>1	94.7%	0.999 (0.997,1)	0.067 (0.063,0.071)	0.994 (0.988,0.998)	0.233 (0.227,0.239)
>2	88.4%	0.996 (0.993,0.997)	0.147 (0.142,0.153)	0.992 (0.987,0.995)	0.147 (0.142,0.153)
>3	81.4%	0.988 (0.984,0.991)	0.235 (0.229,0.242)	0.985 (0.981,0.989)	0.268 (0.262,0.275)
>4	73.7%	0.971 (0.966,0.976)	0.329 (0.322,0.337)	0.976 (0.971,0.98	0.292 (0.284,0.299)
>5	65.2%	0.942 (0.935,0.949)	0.43 (0.422,0.437)	0.963 (0.958,0.967)	0.319 (0.311,0.327)
>6	56.5%	0.891 (0.881,0.90)	0.527 (0.52,0.525)	0.944 (0.94,0.949)	0.349 (0.34,0.357)
>7	47.1%	0.818 (0.806,0.829)	0.628 (0.62,0.635)	0.924 (0.919,0.929)	0.384 (0.375,0.394)
>8	37.7%	0.713 (0.70,0.727)	0.718 (0.711,0.725)	0.898 (0.893,0.903)	0.418 (0.407,0.43)
>9	28.9%	0.599 (0.584,0.613)	0.798 (0.792,0.804)	0.875 (0.87,0.88)	0.457 (0.445, 0.47)
>10	21%	0.466 (0.452,0.481)	0.863 (0.857, 0.868)	0.851 (0.845,0.856)	0.491 (0.476,0.506)
>11	14.6%	0.351 (0.337,0.365)	0.912 (0.908,0.917)	0.832 (0.826,0.837)	0.533 (0.515,0.551)
>12	9.7%	0.252 (0.239,0.264)	0.947 (0.944,0.951)	0.817 (0.811,0.822)	0.575 (0.553,0.597)
>13	6%	0.169 (0.158, 0.18)	0.971 (0.968, 0.973)	0.804 (0.799, 0.81)	0.621 (0.594, 0.648)
>14	3.6%	0.105 (0.965, 0.115)	0.984 (0.982, 0.986)	0.795 (0.789, 0.80)	0.655 (0.619, 0.689)
>15	1.9%	0.06 (0.054, 0.068)	0.992 (0.991,0.994)	0.788 (0.782, 0.794)	0.695 (0.647, 0.74)
>16	1%	0.032 (0.027, 0.038)	0.997 (0.996,0.998)	0.784 (0.778, 0.789)	0.739 (0.672,0.798)
>17	0.4%	0.017 (0.013, 0.021)	0.999 (0.998, 0.999)	0.781 (0.776, 0.787)	0.826 (0.733, 0.897)

.

medRxiv preprint doi: https://doi.org/10.1101/2022.11.06.22281986; this version posted November 7, 2022. The copyright holder for this preprint (which was not certified by peer review) is the author/funder, who has granted medRxiv a license to display the preprint in perpetuity.
It is made available under a CC-BY 4.0 International license .

Supplementary Material 13: Probability of primary adverse outcome for each value of the LMIC-PRIEST score

i) Development cohort (N=305,564)

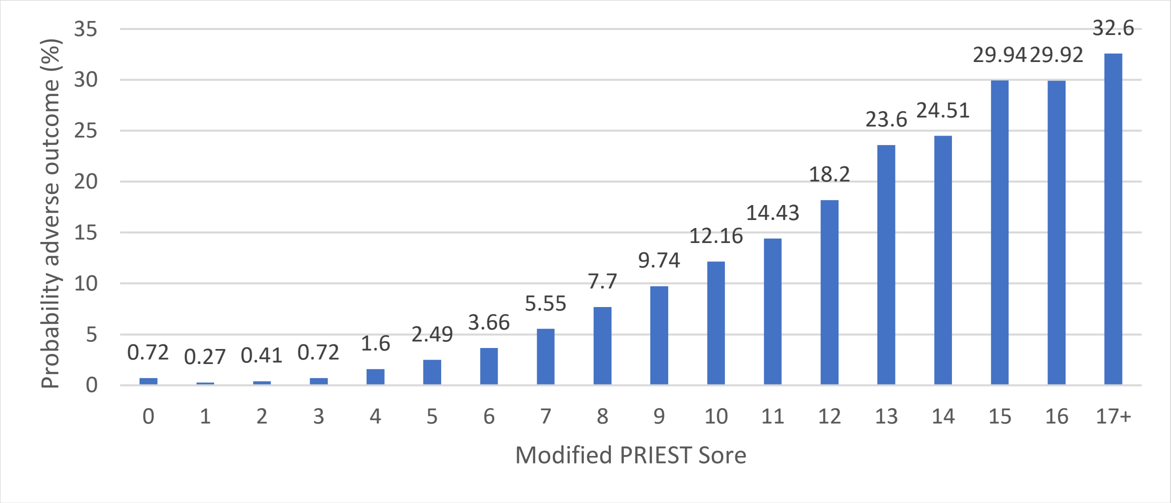

ii) Omicron Validation cohort

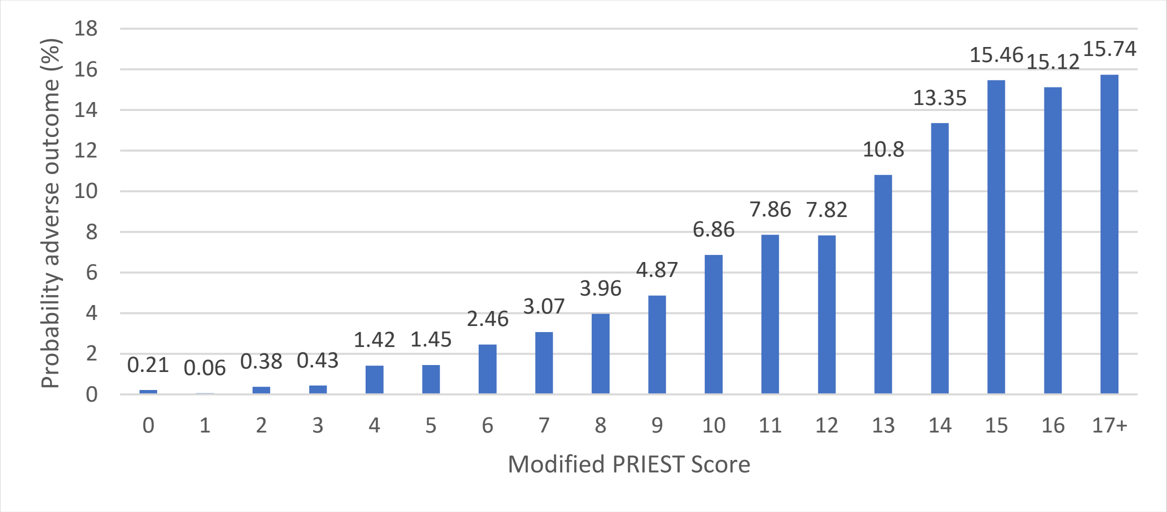

iii) UK PRIEST validation cohort

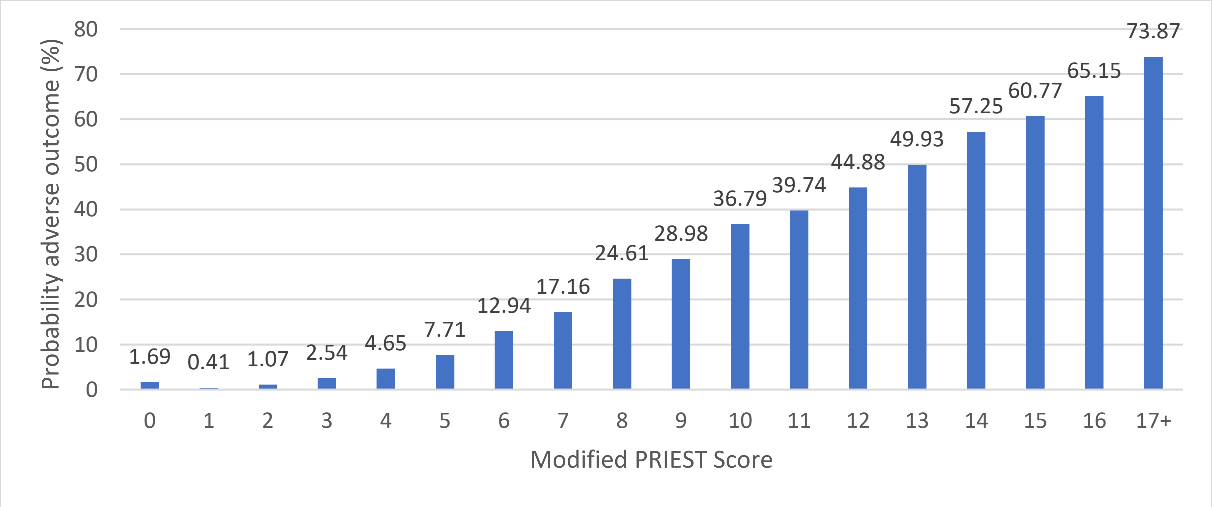

Supplement: 1 [file NIHPP2022.11.06.22281986V1-supplement-1.pdf]
